# Successful Transcatheter Amplatzer Repair of Severe Paravalvular Leak Following Mechanical Mitral Valve Replacement Surgery

**DOI:** 10.1155/cric/9949348

**Published:** 2026-08-19

**Authors:** Akash Ramanathan, Sukesh Burjonroppa, Jeffery Wu, Mohanakrishnan Sathyamoorthy

**Affiliations:** ^1^ Burnett School of Medicine at TCU, Fort Worth, Texas, USA; ^2^ Baylor Heart and Vascular Hospital-Fort Worth Division, Fort Worth, Texas, USA; ^3^ Consultants in Cardiovascular Medicine and Science, Fort Worth, Texas, USA

## Abstract

**Introduction:**

Paravalvular leaks are a rare complication following surgical mitral valve replacement but may result in significant mitral regurgitation. In patients with paravalvular leak, percutaneous repair of the leak is an emerging option for patients who are poor surgical candidates. This report presents a case of a severe symptomatic paravalvular leak postsurgical mitral replacement that was treated with a transcatheter approach resulting in complete resolution of paravalvular regurgitation and symptoms.

**Case Description:**

A 60‐year‐old male with a history of atrial fibrillation, nonischemic cardiomyopathy, Stage III chronic kidney disease, and heart failure with a mildly reduced ejection fraction that improved after undergoing mechanical mitral valve replacement presented with recurrent shortness of breath a few months after surgery. Transthoracic echocardiography demonstrated severe paravalvular leak, with a subsequent TEE confirming the precise etiology and location. After collaborative evaluation in a multidisciplinary valve clinic, the patient underwent successful transcatheter closure with Amplatzer plugs.

**Discussion:**

Transcatheter valve interventions are minimally invasive techniques that have emerged as therapeutic options for patients who are poor surgical candidates. Using this approach to manage PVL is now being studied as a treatment option for patients to avoid redo mitral surgery. This case presents a transcatheter approach to PVL closure in a patient who had undergone a previous surgical mitral valve replacement, resulting in complete resolution of symptoms. The effectiveness of this transcatheter approach signals the need for larger prospective or registry studies to refine transcatheter approaches to manage postsurgical mitral paravalvular leaks.

## 1. Introduction

Mitral paravalvular leaks (PVLs) are a recognized complication that can arise in patients who have undergone prosthetic mitral valve replacements. Mitral PVL is defined as regurgitation from the left ventricle back into the left atrium through a channel between the native annulus and suture ring of the prosthetic mitral valve. PVLs most commonly occur due to dehiscence of the prosthetic sewing ring from the native annulus, but early leaks can result from other technical factors during implantation. Some suggestions for the pathophysiology of mitral PVL are uneven collagen fibers in the posterior aspect of the annulus without a fibrous structure, which predispose it to a PVL [1]. Risk factors that can lead to PVL include prior infective endocarditis, heavy calcification of the annulus, tissue friability, and recent initiation of corticosteroid therapy. The majority of PVLs are likely to occur within the first year of valve implantation, but PVLs developing later must be evaluated for infective endocarditis [2].

Mitral PVLs can occur in 7%–17% of mitral valve replacement procedures [3]. Symptomatic PVL most commonly presents with heart failure manifestations including peripheral edema, fatigue, and dyspnea due to volume overload. Mechanical hemolytic anemia is another manifestation due to the turbulent blood flow which can also contribute to fatigue and dyspnea. Resolution of heart failure symptoms and hemolytic anemia following PVL closure has been associated with improved outcomes [4]. As infective endocarditis is a risk for PVL, patients should be evaluated for signs and symptoms of fevers, chills, night sweats, and peripheral stigmata including splinter hemorrhages, Janeway lesions, and Osler nodes. Diagnosis of mitral PVL requires both a transthoracic echocardiogram and transesophageal echocardiography to evaluate the location and severity of the leak [5]. One study suggests a standardized method of paravalvular regurgitation evaluation for device selection and complete sealing [6].

Current ACC/AHA guidelines recommend surgical closure for patients with hemolytic anemia or heart failure symptoms attributable to prosthetic PVL [7]. For patients at high surgical risk, percutaneous PVL is a reasonable alternative when performed at a comprehensive valve center [7, 8]. The use of transcatheter PVL repairs is becoming an increasingly favorable option due to successful outcomes with lower short‐term mortality [9]. In this report, we present a case of a patient who underwent prosthetic mechanical mitral valve replacement and developed severe paravalvular regurgitation and underwent transcatheter PVL closure using Amplatzer vascular plugs.

## 2. Clinical History

A 60‐year‐old African American male developed highly symptomatic severe mitral regurgitation in 2022 and, after detailed evaluation, underwent surgical, chordal‐sparing replacement with a mechanical valve. Intraoperative TEE demonstrated excellent seating of the valve, normal gradients, and no PVL. He had an uncomplicated postoperative course and was discharged on postoperative Day 7. Approximately 1 month after surgery, he presented to our clinic with progressive shortness of breath with minimal exertion. These symptoms were nearly identical to his clinical picture prior to surgery. He denied fevers, chills, new rashes on extremities, or night sweats.

On cardiovascular exam, there was a normal S1 with physiological split S2 along with a Grade 3–4/6 systolic murmur at the apex that radiated to the axilla. There were no precordial thrills or a displaced point of maximal impulse (PMI). There were no diastolic murmurs noted and no cyanosis or edema on exam. Pulses were 2+ in the carotid, radial, brachial, and dorsalis pedis arteries. On pulmonary exam, there was normal respiratory effort, and lungs were clear to auscultation bilaterally without any wheezes, rales, or rhonchi.

His complete blood count did show signs of anemia with elevated LDH, low haptoglobin, and elevated bilirubin suggestive of hemolytic anemia.

### 2.1. Diagnostic Imaging

#### 2.1.1. Transthoracic Echocardiography

This study revealed an LVEF > 50%, severe left ventricular hypertrophy, and a normal RV size and systolic function without any features of pulmonary arterial hypertension, including an RVSP < 30 mmHg. The mechanical mitral valve was well seated without any rocking motion, but PVL was noted at the septal aspect of the valve. This prompted us to proceed with transesophageal echocardiography to diagnostically characterize the prosthetic mitral valve and severity of PVL.

#### 2.1.2. Transesophageal Echocardiography

**Figure 1:**
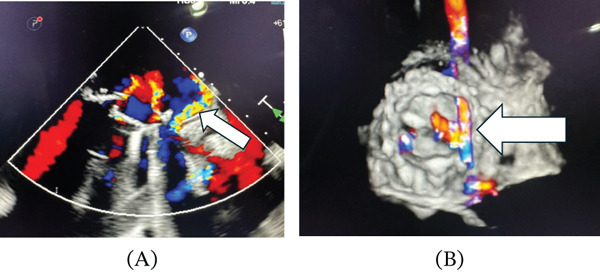
Two‐dimensional and three‐dimensional imaging with color Doppler. (A) TEE imaging at 105° omniplane. (B) 3D TEE imaging. The white arrows point to the PVL in each panel.

Standard TEE 2D and 3D views were utilized. This study revealed a normal ejection fraction > 50%. The mitral valve prosthesis was imaged at 0°, 45°, 75°, 105°, and 135°. The valve was well seated with no rocking motion. There was no heavy calcification (MAC), allowing us to exclude infective endocarditis. There was a normal inward‐directed washing jet and no leaflet dysfunction (Figure 1). There was a severe paravalvular regurgitant jet lateral to the valve along the suture lines representing suture dehiscence. The jet had a Coanda effect wrapping the left atrium. The chordae, papillary muscles, and annulus were all normal. There were no challenging anatomical variations encountered in this patient.

### 2.2. Clinical Decision

We presented this case in a multidisciplinary valve meeting involving echocardiography experts, cardiac surgery, cardiac anesthesiology, and structural heart interventional cardiologists. During the meeting, the patient was deemed to be a poor redo‐operative candidate, and there was consensus that transcatheter Amplatzer plug repair would be favorable given his high‐risk comorbidities, including heart failure with reduced ejection fraction after mitral valve replacement, Stage III chronic kidney disease, atrial fibrillation, and hypertens lamong other comorbidities led to an STS score of operative mortality, 1.65%; morbidity and mortality, 17.5%; stroke, 1.94%; renal failure, 2.87%; reoperation, 5.16%; prolonged ventilation, 11.3%; long hospital stay (> 14 days), 12.6%; and short hospital stay (< 6 days), 17.6%. Although the STS‐predicted risk of operative mortality was low, the composite morbidity was high, with overall surgical risk further elevated by redo sternotomy, frailty, and heart failure with improved ejection fraction. The meeting concluded that since the patient was at elevated surgical risk, a transcatheter approach was deemed appropriate.

### 2.3. Procedure

**Figure 2:**
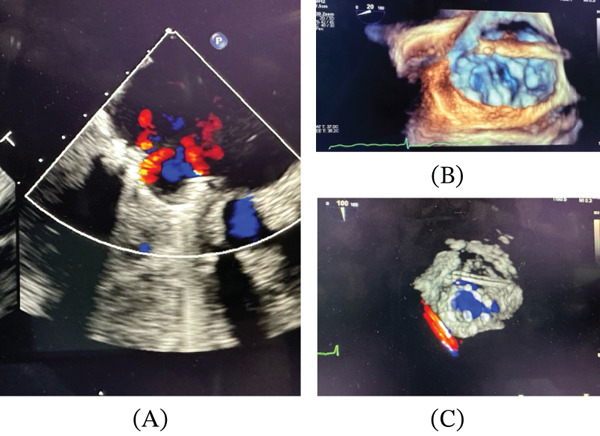
(A) 2D TEE imaging intraprocedure after deployment of the Amplatzer plugs. (B, C) 3D TEE. All panels show complete resolution of the paravalvular leak.

The right common femoral vein was accessed using ultrasound guidance, following which a VersaCross wire was placed in the superior vena cava. Following entry into the right atrium, the fossa ovalis was identified through transesophageal echocardiography prior to access into the left atrium. Access to the left ventricle was achieved through the anteromedial paravalvular defect using a 7‐French internal mammary and 5‐French multipurpose A2 catheter in a mother–child technique with the aid of an angled Glidewire. An 8 mm Amplatzer Vascular Plug II was advanced and deployed with the end lobes straddling the defect in the paravalvular region. A TEE was then used to confirm positioning of the Amplatzer plug along with color Doppler to ensure complete resolution of paravalvular regurgitation. A second leak was noted in the posteromedial area, and another 8 mm Amplatzer Vascular Plug II was advanced and deployed with the end lobes straddling the defect in the paravalvular region. A TEE was again used to confirm the position of the plug along with color Doppler to ensure complete resolution of PVL (Figure 2), with resolution of pulmonary vein S‐dominant flow reversal. The total time of the procedure once vascular access was obtained was 2.5 h, with a fluoroscopy time of 59.1 min. No periprocedure complications occurred.

### 2.4. Outcome

The patient did well posttranscatheter repair and showed marked improvement in clinical follow‐up assessments at 2 weeks, 1 month, and 3 months postimplant. One year postrepair, the patient remains asymptomatic and free from paravalvular regurgitation.

## 3. Discussion

Here, we discuss a patient who underwent a mechanical valve prosthesis that developed a PVL within 1 year of operation. Percutaneous transcatheter closure is an attractive alternative to traditional surgery due to increased risk of adhesions, creating difficulty for surgical field exposure. Recent guidelines consider it acceptable for patients with higher risk heart failure and hemolytic anemia [7]. Specific approaches vary according to leak positioning, the anticipated sizing of the delivery sheath, and the nature of the patient′s aortic/mitral prostheses [5].

In a study involving 139 patients with PVLs, transcatheter closure via the transapical approach (TAp) was performed for a selected 17 high‐risk patients [10]. All patients had a mitral PVL, except one, who had a combined mitral and aortic PVL. The acute procedural success was 98%, and no in‐hospital death occurred in patients who underwent TAp. A retrospective review of 57 percutaneous PVL closures [11] found improvement of at least 1 New York Heart Association (NYHA) functional class in 80% of patients who underwent percutaneous leak closure. The review reported that the percentage of patients requiring blood transfusions and/or erythropoietin injections postprocedure decreased from 56% to 5%. This was further explored by Onorato et al., who showed clinical improvement in heart failure and hemolytic anemia in patients that underwent either mitral valve PVL or aortic valve PVL [12]. Additionally, freedom from cardiac‐related death at 42 months postprocedure was 91.9%. In a study of 381 patients who underwent percutaneous (*n* = 195) or surgical (*n* = 186) treatment of a mitral PVL, technical success was higher in the surgical group (95.5% vs. 70.1%) [13]. However, in‐hospital death occurred in 3.1% and 8.6% of patients undergoing percutaneous and surgical treatment, respectively. A study of 35 patients with mitral PVLs who underwent transcatheter closure notes a success rate above 90% among all leak closure approaches [14]. Furthermore, 77.1% of all patients improved by ≥ 1 NYHA functional class at the 1‐year follow‐up visit, and patients no longer had moderate paravalvular regurgitation during follow‐up examination. These studies highlight and demonstrate safe and effective therapeutic options for PVLs.

Amplatzer vascular plugs are embolic devices that have been used for many different conditions. Originally designed by Dr. Kurt Anton Amplatz for congenital heart defects, it was then approved in 2004 for peripheral vascular embolization. Since then, the use of the Amplatzer plug has expanded to various cardiac procedures [15]. These plugs are made of nitinol braid and function with coils and occlusion balloons to form an embolization device [16]. This device has expanded its use for aortic PVLs which successfully seal leakage around the aortic valve. The success of Amplatzer plugs has been further discussed in studies that showcase the use of multiple plugs for a PVL as an effective and safe method of treatment [17].

In this report, we present a patient who underwent mitral valve replacement for severe mitral regurgitation and subsequently developed severe paravalvular regurgitation within 1 year of surgical replacement, first seen on Doppler echocardiography to evaluate increasing shortness of breath in the postoperative setting. After multidisciplinary evaluation, we determined that the patient was at high surgical risk given his comorbidities and high STS score. We opted to perform a transcatheter deployment of Amplatzer plugs to correct the PVL that was successful and without any complications. After more than 1 year of follow‐up, the patient has done well clinically, demonstrating the effectiveness of our management strategy.

## 4. Conclusion

One unintended and rare outcome of mitral valve replacement is PVL which occurs due to retrograde blood flow into the left atrium through a channel created between the mitral annulus and prosthetic valve, often due to suture dehiscence. Transcatheter closure for PVLs using Amplatzer plugs is an alternative approach for patients that are at high surgical risk. We believe that management in these cases is best achieved through collaborative decision‐making in a multidisciplinary valve clinic involving cardiac surgeons, cardiac anesthesiologists, imaging cardiologists, and structural heart disease interventional cardiologists. This case study highlights the clinical success of the transcatheter approach to manage a PVL and should motivate work in the field to further showcase the success of transcatheter closure with Amplatzer plugs as an effective treatment for patients with PVLs.

## Funding

No funding was received for this manuscript.

## Disclosure

A previous version of this manuscript was submitted in July 2024 on Authorea [18].

## Conflicts of Interest

None of the authors have a conflict of interest to disclose

## Data Availability

The data are not publicly available due to patient privacy considerations.
